# Oxytocin Dosing Intensity and Labor Induction Outcomes by Maternal Body Mass Index

**DOI:** 10.1097/og9.0000000000000150

**Published:** 2026-02-19

**Authors:** Rebecca M. Cohen, Chelsea A. DeBolt, Zhan Zhao, Sara Edwards, Katharine J. McCarthy, Angela Bianco, Luciana Vieira, Ana Capi, Kimberly B. Glazer

**Affiliations:** Icahn School of Medicine at Mount Sinai, New York, New York; Department of Obstetrics, Gynecology and Reproductive Science and Department of Population Health Science and Policy, Icahn School of Medicine at Mount Sinai, New York, New York; and Department of Obstetrics and Gynecology and Department of Biostatistics, Epidemiology and Informatics, University of Pennsylvania Perelman School of Medicine, Philadephia, Pennsylvania.

## Abstract

**OBJECTIVE:**

To examine associations between oxytocin dosing intensity and maternal outcomes—cesarean delivery and postpartum hemorrhage (PPH)—by body mass index (BMI).

**METHODS:**

This retrospective cohort study included singleton births from 2013 to 2022 at a large tertiary hospital among individuals receiving oxytocin for labor induction. Delivery BMI was classified as normal weight (18.5–24.9), overweight (25–29.9), class I–II obesity (30–39.9), and class III obesity (40 or above). Maximum oxytocin dose rate (milliunits per minute) was categorized into quartiles. Modified Poisson regression models, stratified by BMI, estimated risk ratios (RRs) for cesarean delivery and PPH, with adjustment for oxytocin exposure duration, insurance, maternal age, parity, gestational comorbidities, cervical status, gestational age, fetal sex, and birth weight.

**RESULTS:**

Among 20,215 deliveries, 16.9% were to individuals with normal weight, 43.1% to individuals with overweight, 35.1% to individuals with class I–II obesity, and 4.9% to individuals with class III obesity. Maximum oxytocin dose and exposure duration increased with BMI. Individuals in the highest oxytocin quartile (more than 18 milliunits/min) had elevated risk of cesarean delivery and PPH compared with individuals in the lowest two quartiles; associations were partially attenuated after adjustment. Risks associated with highest-quartile dosing were greater for those with normal weight (adjusted RR for cesarean delivery 1.8 [95% CI, 1.5–2.2]; PPH 3.0 [95% CI, 1.7–5.4]) than for those with obesity (cesarean delivery 1.2 [95% CI, 1.0–1.5], class III; PPH 1.6 [95% CI, 1.2–2.0], any class). Among individuals with overweight or obesity, third-quartile dosing was not associated with increased PPH risk.

**CONCLUSION:**

Elevated BMI was associated with greater oxytocin requirements, but risks associated with increased dosing were attenuated in individuals with higher BMI. Tailored dosing strategies may improve labor efficacy and maternal safety.

## Abstract 2

Risks associated with increased oxytocin dosing were attenuated among individuals with higher body mass index. Tailored dosing strategies may improve labor efficacy and safety in this population.

Obesity (body mass index [BMI] 30 or higher) is associated with medical and obstetric complications that may necessitate induction of labor before spontaneous onset.^1–3^ Individuals with obesity are also more likely to experience prolonged pregnancy independently of major comorbidities.^4,5^ As a result, the rate of labor induction increases monotonically with BMI even in otherwise healthy pregnant people.^1,6^ However, elevated BMI is associated with labor dysfunction and delayed progression^7–9^ and is a risk factor for failed induction of labor, resulting in cesarean delivery.^1,10–13^ Intrapartum cesarean delivery is more complicated in individuals with obesity and is associated with higher morbidity compared with either vaginal delivery after induction of labor or prelabor cesarean delivery.^1,7,11,14^

With over 50% of pregnant women in the United States affected by elevated BMI,^13^ optimizing induction protocols to promote vaginal delivery and reduce morbidity is essential. Emerging evidence suggests individuals with obesity may require higher doses of oxytocin to achieve vaginal delivery, potentially because of elevated hormone levels and altered oxytocin diffusion and receptor expression, leading to decreased myometrial contractility.^1,15–19^ Although increased exogenous oxytocin exposure is associated with an increased incidence of peripartum hemorrhage,^19,20^ it remains unclear whether these risks are consistent across BMI categories because they have yet to be assessed by BMI class.

Our study aimed to examine the relationship between oxytocin dose exposure (as determined by maximum dose rate) and rates of cesarean delivery and postpartum hemorrhage (PPH) according to BMI class at delivery. We hypothesized that the association between increased oxytocin exposure and adverse outcomes would be attenuated across increasing BMI categories, reflecting reduced sensitivity to exogenous oxytocin with higher maternal BMI.

## METHODS

**Fig. 1.**
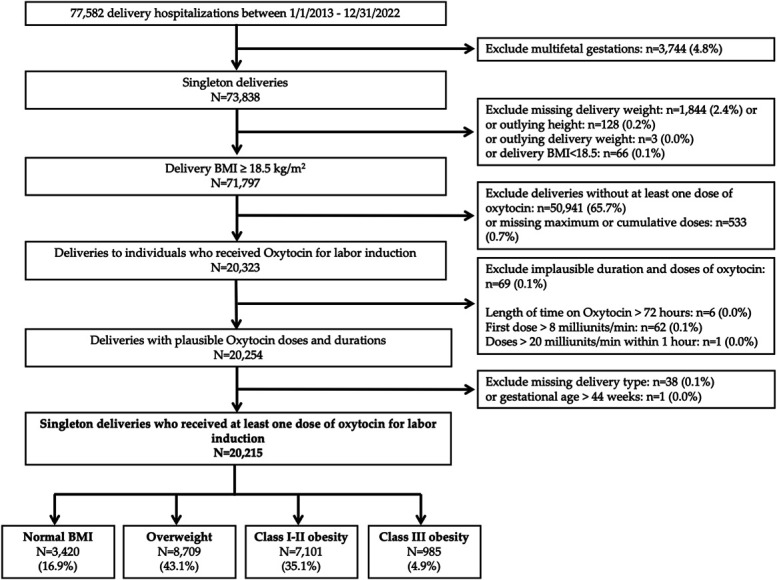
Patient eligibility and exclusion flowchart. Normal weight (18.5–24.9), overweight (25–29.9), class I–II obesity (30–39.9), and class III obesity (40 or above). BMI, body mass index.

We conducted a retrospective cohort study of electronic medical records (EMRs) from all deliveries at a large tertiary hospital in New York City from January 1, 2013, through December 31, 2022 (n=77,582). We first excluded deliveries with multifetal gestations (n=3,744). We further excluded those without a delivery weight within 30 days of delivery (ie, missing delivery weight, n=660), those with outlying height (less than 48 inches or more than 78 inches, n=128), those with outlying delivery weight values (more than 500 lb, n=3), or those classified as underweight based on delivery BMI (BMI below 18.5, n=66) because of small sample size. We then excluded deliveries without exposure to at least one dose of exogenous oxytocin for labor induction (n=50,914) or those with missing maximum or cumulative doses (n=533). Next, we excluded oxytocin administration that was deemed improbable for induction (n=69) based on clinical guidance from study team obstetricians, including cases in which the duration of oxytocin use exceeded 72 hours (n=6), the first dose rate exceeded 8 milliunits/min (n=62), or the dose rate exceeded 20 milliunits/min within 1 hour of the start of the induction (n=1). Lastly, we excluded deliveries with missing delivery type (n=38) and gestational age at birth greater than 44 weeks (n=1). Application of these exclusion criteria resulted in a final study sample of n=20,215 deliveries (Fig. 1). The study was approved by the Icahn School of Medicine at Mount Sinai's Program for the Protection of Human Subjects, and waiver of consent was obtained.

*Cohen. Oxytocin Dosing and Labor Outcomes by BMI. Obstet Gynecol 2026*.

Delivery weight was defined as the self-reported or measured weight recorded in the EMR either at the delivery admission or within 30 days prior, with the value closest to the time of delivery admission used. Consistent with prior literature, delivery BMI was used in this analysis because it reflects maternal physiology at the time oxytocin was administered.^16,20,21^ It was classified according to the World Health Organization: normal weight, 18.5–24.9; overweight, 25–29.9; class I–II obesity, 30–39.9; and class III obesity, 40 or above.^22^

Oxytocin dosing data were obtained from the medication administration record in the institutional EMR. Our institution uses an induction and augmentation of labor protocol for oxytocin dosing for those undergoing a trial of labor. For induction of labor, oxytocin dose rate is initiated at 2 milliunits/min with doubling of the rate every 30 minutes until 8 milliunits/min is reached, after which an incremental increase by 2 milliunits/min every 30 minutes is performed until an adequate contraction pattern is achieved. For augmentation of labor, oxytocin dose rate is initiated at 2 milliunits/min with incremental increase by 2 milliunits/min every 30 minutes until an adequate contraction pattern is achieved. Although the induction protocol is standard, the augmentation protocol may be used according to recommendations from the obstetric team. A dose rate above 30 milliunits/min requires clinical assessment by the obstetric team.

We calculated cumulative oxytocin dose, calculated as the sum of each administered dose multiplied by the time spent at that dose (measured in milliunits), and maximum dose received for each participant. For our main modeling analyses, the primary exposure was maximum oxytocin dose rate in milliunits/minute received before the time of delivery. We focused on maximum dose rate rather than cumulative oxytocin exposure, which conflates duration of labor with pharmacologic effect, to better isolate the association between oxytocin dose intensity and maternal outcomes. We analyzed maximum dose rate as a categorical variable using quartiles based on the distribution in our study population: quartile 1, 0–8 milliunits/min; quartile 2, 9–12 milliunits/min; quartile 3, 13–18 milliunits/min; and quartile 4, more than 18 milliunits/min. We also assessed a binary classification of high-dose oxytocin rate, defined as ever reaching a maximum dose rate of more than 20 milliunits/min over the course of the induction,^23^ or low-dose oxytocin rate. The 20–milliunits/min threshold represents a commonly used intrapartum high-dose rate and has been applied in prior work evaluating obstetric risk associated with high-dose oxytocin exposure.^23^ We measured length of induction as the duration of time on oxytocin, calculated as the difference between the time of delivery and the time of the first dose of oxytocin in the patient's medication administration record.

We evaluated two outcomes of interest: mode of delivery (cesarean versus vaginal delivery) and PPH. Mode of delivery was ascertained as recorded in the delivery summary of the EMR. We identified PPH cases if one of the following criteria was met: 1) documented blood loss exceeding 1,000 mL in the EMR flow sheet, 2) receipt of blood products after the time of delivery, or 3) PPH recorded in the delivery summary.^24^

Covariates, including maternal age, race and ethnicity, and marital status, were obtained from the institutional EMR. We categorized race and ethnicity as non-Hispanic Asian, non-Hispanic Black, Hispanic, non-Hispanic White, and none of the above or unknown race and ethnicity (inclusive of American Indian/Alaska Native, Native Hawaiian/Pacific Islander, EMR-coded categories not listed separately, and unreported race and ethnicity because of the small sample size in each subgroup). Comorbidities such as gestational hypertension, preeclampsia, and gestational diabetes mellitus were identified with International Classification of Diseases, 10th Revision codes (O13, O11, O14, and O24.4). Parity was dichotomized into multiparous and nulliparous. Gestational age at delivery was determined from the best obstetric estimate and categorized according to completed weeks of gestation. Delivery-related variables, including induction method (artificial rupture of membranes, intracervical balloon, oxytocin, and misoprostol), membrane status (spontaneous rupture of membranes or premature rupture of membranes), and cervical status (aggregate Bishop score recorded in the EMR and subcomponents of dilation, effacement, and station), were extracted from either the delivery summary or nursing flowsheet. We used the first recorded value of each measure as the earliest indicator of cervical status on delivery admission. Neonatal data, including Apgar scores and birth weight, were obtained from the EMR delivery summary.

Baseline demographic and maternal pregnancy characteristics were summarized by BMI group. Continuous variables were expressed as mean±SD or median (interquartile range) and compared between groups with *t* tests or Kruskal–Wallis tests. We used χ^2^ tests for bivariate comparisons of categorical measures.

In a first set of descriptive analyses of labor induction trajectories, we examined time to delivery beginning at oxytocin initiation using cumulative incidence plots for cesarean and vaginal delivery, modeled as competing risks and stratified by BMI class. We also plotted the relationship between each participant's maximum and cumulative oxytocin dose received and continuous BMI.

We assessed associations between oxytocin dosing and outcomes (cesarean delivery, PPH) using modified Poisson regression with robust error variance to generate risk ratios (RRs) stratified by BMI group. We included covariates in multivariable regression modes, selected on the basis of a priori hypothesized relationships visualized in a directed acyclic graph, to adjust for confounding bias (Appendix 1, available online at http://links.lww.com/AOG/E522). In the first model, we adjusted for only length of time on oxytocin to distinguish the potential effects of oxytocin dosing intensity (ie, maximum dose reached) independently of induction duration. Next, we additionally adjusted for insurance, maternal age, parity, gestational comorbidities (gestational diabetes or hypertensive disorders of pregnancy), gestational age at delivery, Bishop score closest to delivery admission, fetal sex, and infant birth weight. These covariates were selected from the directed acyclic graph to balance goals of both model parsimony and sufficient adjustment for labor characteristics that may confound associations between oxytocin dosing and delivery outcomes. In a sensitivity analysis, we additionally adjusted for membrane status (categorical variable for artificial rupture of membranes, premature rupture of membranes, spontaneous rupture of membranes) and an indicator variable for the use of more than one induction agent to address potential residual confounding by indication for increased oxytocin dosing. We did not adjust for epidural use because neuraxial anesthesia was used for pain management in over 99.0% of our induction cohort. Models included a robust SE clustered at the patient level to account for multiple deliveries from the same individual across our study period. To assess the potential influence of unmeasured confounding in our study, we calculated E values, which estimate the minimum strength of association that an unmeasured confounder would need to have with both the exposure and the outcome, conditional on the measured covariates, to fully explain away the RRs observed in our study.^25^ In all analyses, values of *P*<.05 were considered significant. We collapsed all obesity classes into one group when stratifying regression models for PPH given sample size constraints.

All statistical analyses and visualizations were performed with R 2024.04.2.

## RESULTS

Of the 20,215 births in our study population, 3,420 (16.9%) were among individuals with normal BMI, 8,709 (43.1%) in those with overweight, 7,101 (35.1%) in those with class I or II obesity, and 985 (4.9%) in those with class III obesity (Fig. 1 and Table 1). Compared with individuals with normal weight, those with higher BMI were more likely to identify as non-Hispanic Black or Hispanic, to have public insurance, and to present with gestational hypertension and gestational diabetes (all *P*<.001). Patients with higher BMI were more likely to receive intracervical balloon, misoprostol, and multiple induction agents. Cervical characteristics, including dilation, effacement, station, and aggregate Bishop score on first cervical examination, were clinically similar, although statistically different, among BMI groups.

**Table 1.** Baseline Characteristics by Body Mass Index Category (n=20,215)

	Total (N=20,215)	Normal Weight (n=3,420)	Overweight (n=8,709)	Obesity Class I and II (n=7,101)	Obesity Class III (n=985)	*P*
Maternal age at delivery, mean±SD (y)	32.8±5.6	32.5±5.6	33.1±5.4	32.8±5.7	31.5±5.9	<.001
Race and ethnicity						<.001
Asian	1,948 (10.0)	492 (14.9)	993 (11.8)	441 (6.4)	22 (2.3)	
Hispanic/Latina	2,077 (10.6)	164 (5.0)	620 (7.4)	1,051 (15.3)	242 (25.3)	
Non-Hispanic Black	1,508 (7.7)	121 (3.7)	399 (4.7)	771 (11.2)	217 (22.7)	
Non-Hispanic White	12,904 (66.0)	2,383 (72.0)	5,960 (70.9)	4,159 (60.4)	402 (42.1)	
None of the above or unknown	1,114 (5.7)	151 (4.6)	431 (5.1)	459 (6.7)	73 (7.6)	
Insurance type						<.001
Commercial	15,905 (78.7)	2,780 (81.3)	7,050 (81.0)	5,399 (76.0)	676 (68.6)	
Public	1951 (9.7)	221 (6.5)	586 (6.7)	920 (13.0)	224 (22.7)	
Self/other	2,359 (11.7)	419 (12.3)	1,073 (12.3)	782 (11.0)	85 (8.6)	
Marital status						<.001
Married/cohabitating	16,506 (81.7)	2,984 (87.3)	7,510 (86.2)	5,447 (76.7)	565 (57.4)	
Single/unknown	3,709 (18.3)	436 (12.7)	1,199 (13.8)	1,654 (23.3)	420 (42.6)	
Parity						.028
Nulliparous	11,982 (60.0)	2067 (61.2)	5,210 (60.5)	4,110 (58.6)	595 (60.8)	
Multiparous	7,998 (40.0)	1,309 (38.8)	3,400 (39.5)	2,905 (41.4)	384 (39.2)	
Hypertensive disorders of pregnancy	2,299 (11.4)	169 (4,9)	725 (8.3)	1,145 (16.1)	260 (26.4)	<.001
Gestational diabetes	1,785 (8.8)	261 (7.6)	639 (7.3)	730 (10.3)	155 (15.7)	<.001
Gestational age, median (IQR) (wk)	39.6 (38.7, 40.4)	39.6 (38.7, 40.4)	39.7 (39.0, 40.6)	39.6 (38.7, 40.4)	39.1 (37.9, 40.3)	<.001
Gestational age (completed wk)						<.001
Less than 37	974 (4.9)	183 (5.5)	384 (4.5)	330 (4.7)	77 (7.9)	
37	1,961 (10.0)	306 (9.2)	709 (8.4)	777 (11.2)	169 (17.4)	
38	2,399 (12.2)	425 (12.8)	943 (11.2)	870 (12.5)	161 (16.5)	
39	6,373 (32.4)	1,101 (33.3)	2,809 (33.2)	2,209 (31.8)	254 (26.1)	
40	4,929 (25.0)	825 (24.9)	2,226 (26.3)	1,684 (24.2)	194 (19.9)	
41 or more	3,045 (15.5)	469 (14.2)	1,380 (16.3)	1,078 (15.5)	118 (12.1)	
Rupture of membrane						
PROM	1939 (10.2)	382 (11.9)	913 (11.2)	572 (8.5)	72 (7.6)	<.001
SROM	3,188 (16.7)	538 (16.7)	1,424 (17.4)	1,094 (16.2)	132 (14.0)	.029
AROM	11,422 (56.5)	1917 (56.1)	4,775 (54.8)	4,109 (57.9)	621 (63.0)	<.001
Cervical characteristics						
Dilation, median (IQR) (cm)	1.0 (0.5, 2.0)	1.0 (0.5, 2.0)	1.0 (0.5, 2.0)	1.0 (0.5, 2.0)	1.0 (0.0, 1.5)	<.001
Effacement, median (IQR) (%)	50.0 (30.0, 60.0)	50.0 (40.0, 70.0)	50.0 (30.0, 60.0)	50.0 (30.0, 50.0)	50.0 (30.0, 50.0)	<.001
Fetal station, median (IQR)	−3.0 (−3.0, −3.0)	−3.0 (−3.0, −2.0)	−3.0 (−3.0, −3.0)	−3.0 (−3.0, −3.0)	−3.0 (−3.0, −3.0)	<.001
Bishop score, median (IQR)	3.0 (2.0, 5.0)	4.0 (2.0, 6.0)	3.0 (2.0, 5.0)	3.0 (2.0, 5.0)	3.0 (1.0, 4.0)	<.001
Induction characteristics						
Intracervical balloon	10,981 (54.3)	1777 (52.0)	4,659 (53.5)	3,927 (55.3)	618 (62.7)	<.001
Misoprostol	1838 (9.1)	251 (7.3)	709 (8.1)	745 (10.5)	133 (13.5)	<.001
More than 1 labor induction agent	14,689 (72.7)	2,433 (71.1)	6,214 (71.4)	5,278 (74.3)	764 (77.6)	<.001
Length of time on oxytocin (hours), median (IQR) (h)	10.7 (6.9, 15.7)	9.4 (6.2, 13.7)	10.4 (6.7, 15.3)	11.4 (7.3, 16.6)	13.8 (9.1, 19.2)	<.001
Newborn characteristics						.314
Apgar score below 7	85 (0.6)	12 (0.5)	32 (0.6)	33 (0.7)	8 (1.1)	

IQR, interquartile range; PROM, premature rupture of membranes; SROM, spontaneous rupture of membranes; AROM, artificial rupture of membranes.

Data expressed as n (%) unless indicated otherwise.

Body mass index cutoffs: normal weight, 18.5−24.9; overweight, 25−29.9; class I–II obesity, 30−39.9; and class III obesity, 40 and above.

The length of time on oxytocin increased with BMI, with the lowest median duration observed among individuals with normal weight (9.4 [interquartile range 6.2, 13.7] hours), followed by those with overweight (10.4 [interquartile range 6.7, 15.3] hours), obesity class I or II (11.4 [interquartile range 7.3, 16.6] hours), and obesity class III (13.8 [interquartile range 9.1, 19.2] hours) (*P*<.001) (Table 1). A positive, monotonic relationship between increasing continuous BMI and maximum oxytocin rate and cumulative oxytocin dose is visualized in Appendixes 2 and 3 (http://links.lww.com/AOG/E522).

**Fig. 2.**
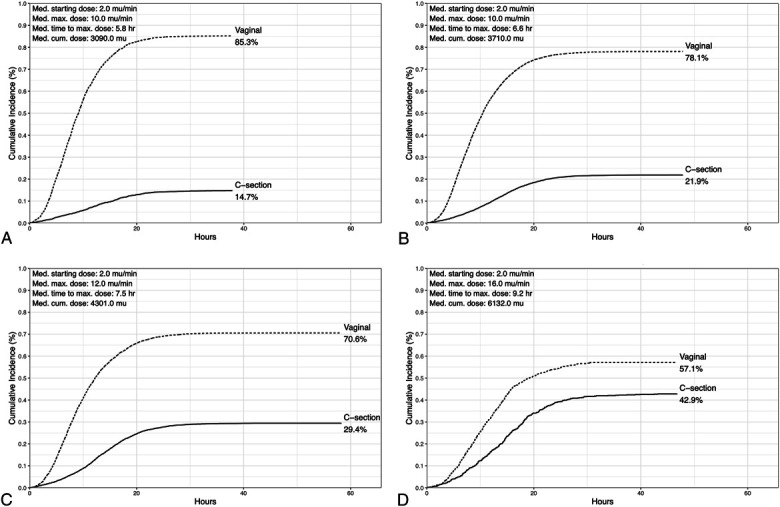
Cumulative incidence rates of vaginal and cesarean delivery among individuals undergoing labor induction with oxytocin by body mass index (BMI) class (n=20,215). Normal weight (18.5–24.9) **(A)**, overweight (25–29.9) **(B)**, class I–II obesity (30–39.9) **(C)**, and class III obesity (40 and above) **(D)**. Med, medium; max, maximum; cum, cumulative.

Overall, 4,924 individuals (24.4%) underwent cesarean delivery (Table 2). Figure 2 illustrates time to vaginal versus cesarean delivery across BMI categories, with slower labor progression and higher rates of cesarean delivery with increasing BMI (14.7%, 21.9%, 29.4%, 42.8% of births for individuals with normal BMI, overweight, obesity class I–II, and obesity class III, respectively). We observed a pattern of increasing maximum oxytocin dose rate, time to reach maximum dose, and greater cumulative dose received across increasing BMI categories (Fig. 2).

**Table 2.** Risk of Cesarean Delivery and Postpartum Hemorrhage Associated With Maximum Oxytocin Dose (Milliunits per Minute) Among Individuals Undergoing Labor Induction Stratified by Maternal Body Mass Index Class (N=20,215)

	Outcome, n, %	Model 1, Unadjusted RR (95% CI)	Model 2, Adjusted RR (95% CI) (Model 1+Duration of Oxytocin Only)	Model 3, Adjusted RR (95% CI) (Model 2+Demographic and Clinical Covariates*)
Cesarean delivery n=4,924 (24.4%)				
Normal weight (n=3,420)	504 (14.7)			
Q1–Q2	208 (10.6)	Reference	Reference	Reference
Q3	92 (10.9)	1.0 (0.8–1.3)	0.8 (0.6–1.0)	0.8 (0.6–1.0)
Q4	204 (33.6)	3.2 (2.7–3.8)	1.8 (1.4–2.2)	1.8 (1.5–2.2)
Overweight (n=8,709)	1,908 (21.9)			
Q1–Q2	619 (14.1)	Reference	Reference	Reference
Q3	375 (16.5)	1.2 (1.0–1.3)	0.9 (0.8–1.1)	0.9 (0.8–1.1)
Q4	913 (44.7)	3.2 (2.9–3.5)	1.9 (1.8–2.2)	1.9 (1.7–2.1)
Obesity class I–II (n=7,101)	2,090 (29.4)			
Q1–Q2	580 (18.4)	Reference	Reference	Reference
Q3	444 (23.4)	1.3 (1.1–1.4)	1.1 (1.0–1.2)	1.0 (0.9–1.2)
Q4	1,066 (52.2)	2.8 (2.6–3.1)	1.9 (1.7–2.1)	1.8 (1.7–2.0)
Obesity class III (n=985)	422 (42.8)			
Q1–Q2	105 (32.2)	Reference	Reference	Reference
Q3	81 (32.0)	1.0 (0.8–1.3)	0.9 (0.7–1.1)	0.9 (0.7–1.1)
Q4	236 (58.3)	1.8 (1.5–2.2)	1.4 (1.1–1.7)	1.2 (1.0–1.5)
Postpartum hemorrhage n=875 (4.3%)				
Normal weight (n=3,420)	100 (2.9)			
Q1–Q2	30 (1.5)	Reference	Reference	Reference
Q3	35 (4.1)	2.7 (1.7–4.4)	2.5 (1.5–4.0)	2.4 (1.4–4.0)
Q4	35 (5.8)	3.8 (2.3–6.1)	3.0 (1.7–5.3)	3.0 (1.7–5.4)
Overweight (n=8,709)	326 (3.7)			
Q1–Q2	125 (2.8)	Reference	Reference	Reference
Q3	73 (3.2)	1.1 (0.9–1.5)	1.0 (0.7, 1.3)	1.0 (0.7–1.3)
Q4	128 (6.3)	2.2 (1.7–2.8)	1.5 (1.1, 2.0)	1.4 (1.0, 1.8)
Obesity class I–III (n=8,086)	449 (5.6)			
Q1–Q2	134 (3.8)	Reference	Reference	Reference
Q3	95 (4.4)	1.1 (0.9–1.5)	1.0 (0.7, 1.3)	0.9 (0.7, 1.2)
Q4	219 (8.9)	2.3 (1.9–2.9)	1.6 (1.2, 2.0)	1.6 (1.2, 2.0)

RR, risk ratio; Q, quartile.

Body mass index classification: normal weight, 18.5–24.9; overweight, 25–29.9; class I–II obesity, 30–39.9; and class III obesity, 40 or above.

Maximum dose quartile cutoffs: Q1–Q2, 0–12 milliunits/min; Q3, 13–18 milliunits/min; and Q4, more than 18 milliunits/min.

* Adjusted for length of time on oxytocin plus insurance coverage, maternal age, parity, hypertensive disorders of pregnancy or gestational diabetes, Bishop score, fetal sex, gestational age at delivery, and birth weight.

*Cohen. Oxytocin Dosing and Labor Outcomes by BMI. Obstet Gynecol 2026*.

In unadjusted analyses, individuals with a maximum oxytocin dose rate in the highest quartile (quartile 4) had a higher risk of cesarean delivery compared with those in the lowest two quartiles (quartiles 1–2) (model 1, Table 2). Associations remained after adjustment for total duration of oxytocin exposure, although effect sizes were attenuated (model 2, Table 2). Further adjustment for maternal sociodemographic and clinical characteristics—including insurance, maternal age, parity, gestational comorbidities, gestational age at delivery, Bishop score closest to delivery admission, fetal sex, and infant birth weight—did not notably change effect estimates (model 3, Table 2). The RRs for highest-quartile dosing were weaker among individuals with class III obesity compared with individuals in other BMI categories. A maximum dosing rate in quartile 3 was not associated with increased risk of cesarean delivery in any BMI group. Similar patterns were observed when oxytocin exposure was dichotomized as high (more than 20 milliunits/min) versus low (20 milliunits/min or less) dose (Appendix 4, http://links.lww.com/AOG/E522).

Postpartum hemorrhage occurred in 4.3% of deliveries (n=875), with increasing prevalence across BMI categories: 2.9% among individuals with normal weight, 3.7% among those with overweight, and 5.6% among those with obesity (any class) (Table 2). In unadjusted models, individuals who reached a quartile 4 maximum oxytocin dosing rate had greater risk of PPH compared with those in quartiles 1 and 2 across all BMI groups: normal weight (RR 3.8 [95% CI, 2.3–6.1]), overweight (RR 2.2 [95% CI, 1.7–2.8]), and obesity class I–III (RR 2.3 [95% CI, 1.9–2.9]). Associations remained but were attenuated with adjustment (models 2 and 3, Table 2). The risk of PPH associated with highest-quartile dosing was greater for individuals with normal weight (adjusted RR 3.0 [95% CI, 1.7–5.4]) than for those with overweight (1.4 [95% CI, 1.0–1.8]) or obesity (1.6 [95% CI, 1.2–2.0]). In both unadjusted and adjusted models, third-quartile dosing was associated with higher risk of PPH among individuals with normal weight but was not associated with PPH among those with overweight or obesity.

In dichotomous high- versus low-dose analyses, receipt of high-dose oxytocin was associated with increased PPH among individuals with overweight and obesity but not those with normal weight, although analyses in the normal-weight category included very few individuals with high-dose oxytocin (Appendix 4, http://links.lww.com/AOG/E522).

For both cesarean delivery and PPH, the associations across all three models demonstrated reasonable robustness to unmeasured confounding (Appendix 5, http://links.lww.com/AOG/E522).

In addition, in both cesarean delivery and PPH analyses, RRs were the same after models were further adjusted for membrane status and use of multiple induction agents, as well as in models that were subset to term births.

## DISCUSSION

In this study of more than 20,000 labor inductions, both oxytocin dosing and duration of exposure increased with BMI class, as did rates of cesarean delivery and PPH. Individuals receiving oxytocin doses in the highest quartile (more than 18 milliunits/min) had elevated risk of cesarean delivery and PPH compared with individuals in the lowest quartiles. These associations remained but were markedly attenuated after adjustment for oxytocin duration and patient characteristics. Among patients with overweight and obesity, receiving a third-quartile maximum dose was not associated with increased risk of cesarean delivery or PPH.

Our findings reinforce the links between higher BMI and risk of cesarean delivery and PPH,^2–4,6,10,21,26^ particularly in induction cohorts,^1,10,14^ and further demonstrate greater oxytocin requirements and longer induction durations compared with those with normal BMI.^1,9,13,15,16,21^ Physiologic mechanisms underlying these higher oxytocin requirements have not been elucidated fully but include altered oxytocin receptor expression, disrupted calcium signaling, and impaired oxytocin diffusion necessitating higher doses to reach effective concentration at myometrial receptor sites.^15–19^

Although individuals with elevated BMI may have a higher physiologic requirement for exogenous oxytocin, excessive dosing may increase the risk of adverse maternal outcomes.^26,27^ Whereas some studies have found associations between high oxytocin dosing and cesarean delivery^23^ or PPH,^23,28^ these often attenuate after adjustment for covariates, including BMI^28^ and intrapartum oxytocin duration.^23^ In stratified analyses, we found that the highest-quartile dosing rate was associated with increased cesarean delivery and PPH in all BMI classes but with evidence of weaker associations with higher BMI, substantial attenuation of associations after adjustment for duration of oxytocin exposure, and minimal additional change with inclusion of other patient-level covariates. Our findings highlight the importance of disentangling the potential effects of dosing intensity (ie, maximum dose rate) independently of dose duration in the study of outcomes of labor induction.^29^ Although both are components of total oxytocin exposure, they may have distinct physiologic and clinical implications for labor progression. Prior studies have rarely explored this distinction, and to the best of our knowledge, none have examined whether the relationship between oxytocin dosing characteristics and delivery outcomes varies across BMI classifications.

By stratifying by BMI instead of adjusting for it as a confounder, we explicitly modeled oxytocin–outcome relationships within BMI categories. This approach is critical to inform individualized practice guidelines for patients with elevated BMI given well-established BMI-based differences in labor course and outcomes. For example, individuals with overweight or obesity had roughly half the risk of PPH associated with highest-quartile dosing as those with normal weight, and third-quartile dosing (12–18 milliunits/min) was not associated with either cesarean delivery or PPH among individuals with overweight or obesity. These results suggest dosing ranges that may represent a potential target for future safety and efficacy studies to improve the outcomes of labor induction among individuals with elevated BMI.

Although our population included deliveries within a single health system, limiting generalizability, studying births within a system with a standardized induction protocol reduced provider-level practice variation that may otherwise influence results. We acknowledge the potential for residual confounding by cesarean delivery indication or PPH cause, whereby observed associations may reflect underlying patient characteristics prompting intervention rather than the effects of the intervention itself. To address this, we used causal diagrams to select sociodemographic and obstetric covariates that may influence oxytocin dosing for inclusion in our main analyses. We examined the robustness of our adjusted estimates to residual confounding in sensitivity analyses restricted to term births and controlling for additional characteristics. Furthermore, we found that fairly strong unmeasured residual confounding would be required to nullify the associations found in our study. However, we lacked information on prior cesarean delivery, induction indication, cesarean delivery indication, PPH cause, uterine contractility, fetal tracing, a full range of maternal comorbidities, and newborn outcomes. Future research should include time-varying data on additional intrapartum and maternal–neonatal outcomes to suggest tailored induction strategies in populations with high BMI. In addition, comparisons between spontaneous labor onset and induction of labor across BMI categories may help elucidate differences in oxytocin exposure that contribute to disparities in labor outcomes.

Our study has notable strengths. We leveraged detailed EMR data from a tertiary care health system, allowing us to capture granular clinical variables, including date and time-stamped medication administration, which is not available in many secondary databases. Our large sample size, including 10 years of deliveries, enabled stratified analyses by BMI, oxytocin exposure levels, and analysis of PPH, which is a clinically significant adverse outcome. Compared with existing research,^20,30^ our cohort included a relatively large population of individuals with class III obesity, an increasing but understudied population in obstetric research.

In summary, in a cohort with more than 20,000 labor inductions, elevated BMI was associated with greater oxytocin requirements. However, risks associated with higher dosing were attenuated among individuals with higher BMI. Future prospective studies focused on individualized induction protocols are needed to establish optimal dosing strategies that balance efficacy and maternal safety, particularly among patients with elevated BMI.
